# Wave propagation and thermal behavior in nonlocal thermoelastic porous media under moving heat sources with three-phase-lag and Green–Naghdi models

**DOI:** 10.1038/s41598-026-50607-x

**Published:** 2026-05-18

**Authors:** Mohamed I. A. Othman, Samia M. Said, Esraa M. Gamal

**Affiliations:** Department of Mathematics, Faculty of Science, Zagazig University, Zagazig, 44519 Egypt

**Keywords:** Porous thermoelastic solid, Moving heat source, Nonlocal parameter, Three-phase-lags model, Green-Naghdi theory, Normal mode analysis, Engineering, Materials science, Mathematics and computing

## Abstract

This study examines thermoelastic wave propagation in a nonlocal porous thermoelastic half-space subjected to a moving heat source using two advanced theoretical models: the three-phase-lag (3PHL) heat conduction model and the Green–Naghdi type III theory. Analytical solutions for the physical field variables are derived via normal mode analysis. The work highlights the role of nonlocal effects in porous media and presents a comparative assessment of the two thermoelastic frameworks. Numerical results demonstrate that locality reduces displacement and stress amplitudes, while porosity and phase-lag parameters significantly influence temperature and volume fraction fields. Additionally, the moving heat source modifies wave propagation characteristics and enhances the thermal response near the boundary. These results offer valuable insight into thermally induced deformation in porous materials and are relevant to applications such as laser processing, additive manufacturing, and thermal protection systems.

## Introduction

The generalization of thermoelasticity is known as the thermoelasticity of dual-phase-lag, developed by Tzou^1^ and Chandrasekhariah^2^. Tzou^1^ introduced the concept of microstructural effects by considering time delay in the macroscopic response, attributing the lattice temperature delay to the photon-electron interaction at the macroscopic level. To address relevant heat transfer problems involving very short time intervals and precise heat fluxes, the hyperbolic equation yields results that differ from the parabolic equation. Roy Choudhuri^3^ extended this further by introducing the three-phase-lag (3PHL) model. Many surveys that highly spot the advantageous consequences in terms of postulation of the 3PHL hypothesis have been published. Kumar and Mukhopadhyay^4^ focused on the impact of the three-phase-lag (3PHL) model on thermoelastic interactions, specifically examining the effect of a step temperature signal at the stress-free boundary of a cylindrical cavity in an infinite medium. Abbas^5^ investigated fractional-order generalized thermoelasticity in a functionally graded medium using the 3PHL model under thermal shock conditions. Marin et al.^6^ explored a mixed initial-boundary value problem by modeling a 3PHL dipolar thermoelastic body. The thermoelastic solid hypothesis via the 3PHL was drawn out as^7–12^.

The poro-thermoelasticity theory is crucial for analyzing porous materials in geotechnical engineering, particularly in understanding how soil and rock respond to thermal and mechanical stresses, fluid flow, and heat transfer. The sphere of impact and uniqueness that lead to thermoelastic solids with voids were examined by Marin^13,14^. The distortion resulting from agitated loads in a thermoelastic solid with pores was first described by Kumar and Rani^15^. Sharma and Kumar^16^ showed the extension of plane waves in a thermo-viscoelastic material with pores. Aouadi et al.^17^ explored a nonlinear model of thermoelasticity in porous materials and diffusion, in accordance with the Green–Naghdi thermo-mechanical theory of types II and III. Fahmy^18^ examined the effects of material arrangement and rotation on the optimal behavior of rotating visco-elastic porous semi-permeable structures. Żur et al.^19^ unaddressed the nonlinear dynamics of porous composite nanobeams joined with fullness. Fernández and Quintanilla^20^ studied a porous thermoelastic problem with microtemperatures assumptive parabolic higher order in time derivatives for the thermal variables. Elzayady et al.^21^ provided a theoretical examination of porous elastic solids enclosed by a magnetic field exploring the dual phase lag hypo-thesis. A poro-thermoelastic material issue with reference temperature and an inclined load is presented by Said^22^. Some new contributions to porous media under the influence of external parameters have been discussed in^23–25^.

Das and Kanoria^26^ investigated a magneto-thermoelastic interaction problem in a functionally graded material subjected to periodically varying heat sources. Sarkar and Lahiri^27^ presented an issue with a thermoelastic solid capable of a moving heat source on the extremity of space, which is traction escape. Ailawalia and Singla^28^ displayed the disturbance owing to a moving heat source in thermoelastic material using a dual phase lag hypothesis. Abbas^29^ employed the eigenvalue approach to solve a problem based on the fractional-order magneto-thermoelastic theory involving a moving heat source plane. Jain et al.^30^ discussed the contact of a heat source on a fractional-order fiber-reinforced thermoelastic solid. Youssef and Al-Lehaibi^31^ showed a problem of a three-dimensional thermoelastic solid capable of a moving heat source. Zenkour et al.^32^ presented the coupled thermoelastic response of an infinite medium containing a cylindrical cavity subjected to a moving heat source. Das et al.^33^ investigated a magneto-thermoelastic problem in a half-space under the influence of heat flux applied at the boundary surface. Said^34^ displayed a problem of a nonlocal thermoelastic solid with a moving internal heat source caused by gravitational force and mechanical force. The study conducted by Ailawalia et al.^35^ explores the behavior of an infinite thermo-elastic plate with an internal heat source, analyzed within the framework of Moore–Gibson–Thompson (MGT) theory, while being subjected to the influence of viscous fluid layers.

From an engineering perspective, the analysis of thermoelastic media subjected to moving heat sources is of significant importance in several modern applications. Such configurations arise naturally in laser surface treatment, additive manufacturing processes, and thermal barrier coating technologies, where localized heat sources move relative to the material surface. In these applications, porous materials are frequently employed due to their lightweight structure and enhanced thermal insulation properties. The incorporation of nonlocal thermoelastic effects allows for a more accurate description of stress-smoothing and size-dependent behavior at micro- and nano-scales, which cannot be captured by classical local theories. Therefore, the combined consideration of nonlocality, porosity, and moving heat sources provides a more realistic framework for modeling heat-induced deformation in advanced engineering materials.

The originality of this research lies in the development of a novel model for the linear analysis of a nonlocal porous thermoelastic medium subjected to a moving heat source, based on the three-phase-lag (3PHL) model and the Green–Naghdi type III (G-N III) theory. Unlike classical local models, the formulation incorporates nonlocal elasticity, allowing the modeling of long-range interactions and size-dependent behavior, which is particularly relevant for micro-structured or nano-engineered porous materials. Closed-form analytical solutions are obtained using the normal mode analysis technique, enabling a systematic evaluation of the influence of nonlocality, porosity, thermal relaxation, and heat source motion on the coupled physical fields. Moreover, a dual-model approach provides a comprehensive comparison of the thermoelastic responses predicted by the 3PHL and G-N III theories under identical loading and material conditions, offering insight into the strengths and limitations of each framework. Collectively, these contributions advance the understanding of thermoelastic wave behavior in porous media under dynamic thermal environments and provide a foundation for future studies and engineering applications.

## Formulation of the issue

An isotropic, homogeneous, thermoelastic porous solid is considered, subjected to a moving heat source. We take the rectangular Cartesian coordinates with origin on the surface $z=0$ and $x$-axis normally into the medium, which is represented by $x \geqslant 0$. The displacement vector is given by $u=u(x,y,\,t),\,\,\,\,v=v(x,y,\,t),\,\,\,w=0,\,\,\,\,\frac{\partial }{{\partial \,z}}=0\,.$

The constitutive equations like Said^22^ and Eringen^36^

1 $$(1 - {\varepsilon ^2}\,{\nabla ^2})\,{\sigma _{ij}}=\lambda \,{e_{kk}}{\delta _{ij}}+2 \mu \,{e_{ij}} - \gamma \,\theta \,{\delta _{ij\,}}+b\,\psi {\delta _{ij\,}}.$$

Roy Choudhuri’s^3^ formulation of the heat conduction equation.

2 $$\,K{\,^*}\,{\nabla ^2}\theta +\tau _{\nu }^{*}\,{\nabla ^2}{\theta _{,t}}+K\,{\tau _\theta }{\nabla ^2}{\theta _{,tt}}=(1+{\tau _q}\frac{\partial }{{\partial t}}+\frac{1}{2}\tau _{q}^{2}\frac{{{\partial ^2}}}{{\partial {t^2}}})\,(\rho \,{C_E}{\theta _{,tt}}+\gamma \,{T_0}\,{e_{,tt}}+{\alpha _3}{T_0}{\psi _{,tt}} - Q).$$

The motion equations as^23^

3 $$\rho \,{\ddot {u}_i}={\sigma _{ji,\,j}}$$

The equation of voids as^22^

4 $$\rho \,{\alpha _4}(1 - {\varepsilon ^2}\,{\nabla ^2})\,{\psi _{,tt}}=\beta {\psi _{ii}} - b\,e - {\alpha _1}\,\psi - {\alpha _2}\,{\psi _{,t}}+{\alpha _3}\theta .$$

Initiate Eq. (1) in Eq. (3), we get

5 $$\rho \,(1 - {\varepsilon ^2}{\nabla ^2})\,\,{u_{,tt}}=\,{B_{\,1}}\,\frac{{{\partial ^{\mathrm{2}}}u\,}}{{\partial \,{x^{\mathrm{2}}}}}+{B_{\,2}}\,\frac{{{\partial ^{\mathrm{2}}}v\,}}{{\partial \,x\,\,\partial \,y\,}}+{B_{\,3}}\,\frac{{{\partial ^{\mathrm{2}}}u\,}}{{\partial \,{y^{\mathrm{2}}}}} - \gamma \frac{{\partial \,\theta \,}}{{\partial \,x}}+\,b\,\,\frac{{\partial \,\psi \,}}{{\partial \,x}},$$

6 $$\rho \,(1 - {\varepsilon ^2}{\nabla ^2})\,\,{v_{,tt}}=\,{B_{\,3}}\,\frac{{{\partial ^{\mathrm{2}}}v\,}}{{\partial \,{x^{\mathrm{2}}}}}+{B_{\,2}}\,\frac{{{\partial ^{\mathrm{2}}}u\,}}{{\partial \,x\,\,\partial \,y\,}}+{B_1}\,\frac{{{\partial ^{\mathrm{2}}}v\,}}{{\partial \,{y^{\mathrm{2}}}}} - \gamma \frac{{\partial \,\theta \,}}{{\partial \,y}}+\,b\,\,\frac{{\partial \,\psi \,}}{{\partial \,y}},$$

where $${B_1}=\lambda +2\mu ,\,\,\,{B_2}=\lambda +\mu ,\,\,\,{B_3}=\mu$$.

For suitableness, the pursuing non-dimensional variables are introduced:

7 $$\begin{aligned} (x^{\prime } ,y^{\prime } ,\varepsilon ^{\prime } ,) & = \frac{{\omega _{{{\kern 1pt} 1}} }}{{c_{1} }}(x,y,\varepsilon ),(u^{\prime } ,v^{\prime } ) = \frac{{\rho \omega _{1} c_{1} }}{{\gamma T_{0} }}(u,v),\theta ^{\prime } = \frac{\theta }{{T_{0} }},Q^{\prime } = \frac{1}{{\lambda \omega _{1} }}Q,\psi ^{\prime } = \frac{{\rho c_{1}^{2} }}{{\gamma T_{0} }}\psi , \\ & \sigma ^{\prime } _{{ij}} = \frac{{\sigma _{{ij}} }}{{\gamma T_{0} }},(t^{\prime } ,\tau ^{\prime } _{q} ,\tau ^{\prime } _{\theta } ,\tau ^{\prime } _{\nu } ){\mkern 1mu} = \omega _{1} (t,{\mkern 1mu} \tau _{q} ,{\mkern 1mu} \tau _{\theta } ,\tau _{\nu } ),c_{1}^{2} = \frac{{\lambda + 2\mu }}{\rho },\omega _{1} = \frac{{\rho {\mkern 1mu} C_{E} c_{1}^{2} }}{K}. \\ \end{aligned}$$

Initiate Eq. (7) in Eqs. (2) and (4)–(6), we get

8 $$(1 - {\varepsilon ^2}{\nabla ^2})\,\,{u_{,tt}}=\,{h_1}\,\frac{{{\partial ^{\mathrm{2}}}u\,}}{{\partial \,{x^{\mathrm{2}}}}}+{h_2}\,\frac{{{\partial ^{\mathrm{2}}}v\,}}{{\partial \,x\,\,\partial \,y\,}}+{h_3}\,\frac{{{\partial ^{\mathrm{2}}}u\,}}{{\partial \,{y^{\mathrm{2}}}}} - \frac{{\partial \,\theta \,}}{{\partial \,x}}+\,{h_4}\,\,\,\frac{{\partial \,\psi \,}}{{\partial \,x}},$$

9 $$(1 - {\varepsilon ^2}{\nabla ^2})\,\,{v_{,tt}}=\,{h_3}\,\frac{{{\partial ^{\mathrm{2}}}v\,}}{{\partial \,{x^{\mathrm{2}}}}}+{h_2}\,\frac{{{\partial ^{\mathrm{2}}}u\,}}{{\partial \,x\,\,\partial \,y\,}}+{h_1}\,\frac{{{\partial ^{\mathrm{2}}}v\,}}{{\partial \,{y^{\mathrm{2}}}}} - \frac{{\partial \,\theta \,}}{{\partial \,y}}+{h_4}\,\,\frac{{\partial \,\psi \,}}{{\partial \,y}},$$

10 $${a_5}(1 - {\varepsilon ^2}\,{\nabla ^2})\,{\psi _{,tt}}={\psi _{ii}} - {a_1}\,e - {a_2}\,\psi - {a_3}\,{\psi _{,t}}+{a_4}\theta ,$$

11 $${d_1}\,{\nabla ^2}\theta +{d_2}{\nabla ^2}\dot {\theta }+{d_3}{\nabla ^2}{\theta _{,tt}}=(1+{\tau _q}\frac{\partial }{{\partial t}}+\frac{1}{2}\tau _{q}^{2}\frac{{{\partial ^2}}}{{\partial {t^2}}})\,({\theta _{,tt}}+{d_4}\,{e_{,tt}}+{d_5}{\psi _{,tt}} - {d_6}Q),$$

$$where,\,\,\,({h_1},\,{h_2},\,{h_3},\,{h_4},\,{h_5})=\,\,(\frac{{{B_1}}}{{\rho \,c_{1}^{2}}},\,\frac{{{B_2}}}{{\rho \,c_{1}^{2}}},\,\frac{{{B_3}}}{{\rho \,c_{1}^{2}}},\frac{b}{{\rho \,c_{1}^{2}}},\,\frac{\lambda }{{\rho \,c_{1}^{2}}}),\,\,\,\,\,{a_1}=\frac{{b\,c_{1}^{2}}}{{\beta \,\omega _{1}^{2}}},\,\,\,\,\,\,{a_2}=\frac{{{\alpha _1}\,c_{1}^{2}}}{{\beta \,\omega _{1}^{2}}},$$

$${a_3}=\frac{{{\alpha _2}\,\,c_{1}^{2}}}{{\beta \,{\omega _1}}},\,\,\,\,{a_4}=\frac{{{\alpha _3}\,\,\rho \,c_{1}^{4}}}{{\beta \,\gamma \,\omega _{1}^{2}}},\,\,\,\,\,{a_5}=\frac{{\rho \,{\alpha _4}\,\,c_{1}^{2}}}{{\beta \,}},\,\,\,\,{d_1}=\frac{{{K^*}}}{{\rho \,{C_E}\,c_{1}^{2}}},\,\,\,\,{d_2}=1+{d_1}{\tau _\nu },\,\,\,\,{d_3}={\tau _\theta },\,$$

$${d_4}=\frac{{{\gamma ^2}\,{T_0}}}{{{\rho ^2}\,{C_E}\,c_{1}^{2}}},\,\,\,\,{d_5}=\frac{{{\alpha _3}\gamma \,{T_0}}}{{\rho \,{C_E}\,c_{1}^{2}\,}},\,\,\,\,\,{d_6}=\frac{\lambda }{{\,\rho \,{C_E}\,{T_0}{\omega _1}}}.\,$$

## Normal mode analysis

We apply the normal mode analysis method to obtain the solution as Said^29^:

12 $$(\,u,v,\,\theta ,\,\psi ,{\sigma _{ij}}\,)\,\,(x,y,t)=(\,\bar {u},\bar {v},\,\bar {\theta },\,\bar {\psi },{\bar {\sigma }_{ij}}\,)(x)\exp \,({\mathrm{i}}\,ry - mt)\,,$$

13 $$Q=\bar {Q}\exp (iry - mt),\,\bar {Q}={\bar {Q}_0}{v_0}$$

where $\bar {u}(x)$ and so on, denote the amplitude functions of $u\,(x,y,\,t)$, and related variables. Normal mode analysis is, in fact, to look for the solution in a Fourier transformed domain, assuming that all the field quantities are sufficiently smooth on the real line such that normal mode analysis of these functions exists.

Exploitation Eqs. (12) and (13) in Eqs. (8)–(11), we get

14 $$({A_{\,1}}{{\mathrm{D}}^2} - {A_{\,2}})\,\,\bar {u}+{\mathrm{i}}r{h_{\,2}}{\mathrm{D}}\,\bar {v} - {\mathrm{D}}\bar {\theta }+{h_4}\,{\mathrm{D}}\,\bar {\psi }=0,$$

15 $${\mathrm{i}}r{h_{\,2}}{\mathrm{D}}\bar {u}+\,({A_3}{{\mathrm{D}}^2} - {A_4})\,\bar {v} - {\mathrm{i}}r\bar {\theta }+{\mathrm{i}}r{h_4}\,\bar {\psi }=0,$$

16 $${a_{\,1}}{\mathrm{D}}\,\bar {u}+{\mathrm{i}}{a_1}\,r\,\bar {v} - {a_4}\bar {\theta }+({A_5}\, - {A_6}{{\mathrm{D}}^2}\,)\,\bar {\psi }=0,$$

17 $${A_8}{\mathrm{D}}\bar {u} - {A_9}\bar {v}+({A_{10}}{{\mathrm{D}}^2} - {A_{11}})\,{\text{ }}\bar {\theta }+{A_{12}}\bar {\psi }={A_{13}}{Q_0}{v_0},$$

$$where\,\,\,\,{A_1}={h_1}+{\varepsilon ^2}{m^2},\,\,\,\,\,\,\,{A_2}={h_3}\,{r^2}+{m^2}+{\varepsilon ^2}{m^2}{r^2},\,\,\,\,\,\,\,{A_3}={h_3}+{\varepsilon ^2}{m^2},$$

$${A_4}={h_1}\,{r^2}+{m^2}+{\varepsilon ^2}{m^2}{r^2},\,\,\,\,\,{A_5}=\,{r^2}+{a_2} - {a_3}m+{a_5}{m^2},\,\,\,\,\,\,\,{A_6}=1+{a_5}{\varepsilon ^2}{m^2},$$

$$A_{{11}} = d_{1} r^{2} - d_{2} mr^{2} + d_{3} m^{2} r^{2} + A_{7} m^{2} ,\,\,A_{{12}} = d_{5} m^{2} A_{7} ,\,\,A_{{13}} = d_{6} A_{7} ,\,\,D = \frac{d}{{d\,x}}$$

The scheme of Eqs. (14)–(17) is resolved to get:

18 $$({{\mathrm{D}}^{\mathrm{8}}} - {E_1}{{\mathrm{D}}^{\mathrm{6}}}+{E_2}{{\mathrm{D}}^{\mathrm{4}}} - {E_3}\,{{\mathrm{D}}^{\mathrm{2}}}+{E_4})\bar {u}(x)=0,$$

$$where,\,\,\,\,{E_1}=\frac{{{A_{15}}+{A_{16}}}}{{{A_{\,14}}}},\,\,\,\,{E_2}=\frac{{{A_{17}}+{A_{18}}+{A_{19}}+{A_{20}}}}{{{A_{\,14}}}},\,\,\,\,{E_3}=\frac{{{A_{21}}+{A_{22}}+{A_{23}}+{A_{24}}+{A_{25}}}}{{{A_{\,14}}}},$$

$${E_4}=\frac{{{A_{26}}+{A_{27}}}}{{{A_{\,14}}}},\,\,\,\,{A_{\,14}}={A_1}{A_3}{A_6}{A_{10}},\,\,\,{A_{\,15}}={A_1}{A_3}{A_5}{A_{10}} - {r^2}{A_6}{A_{10}}\,h_{2}^{2} - {A_3}{A_6}{A_8},$$

$${A_{16}}={A_1}{A_3}{A_6}{A_{11}}+{A_1}{A_4}{A_6}{A_{10}}+{A_2}{A_3}{A_6}{A_{10}} - {a_1}{h_4}{A_3}{A_{10}},$$

$${A_{17}}={a_1}{A_3}{A_{12}} - {A_4}{A_6}{A_8} - {A_3}{A_5}{A_8}+{A_1}{A_3}{A_5}{A_{11}}+{A_1}{A_4}{A_5}{A_{10}},$$

$${A_{18}}={A_2}{A_3}{A_5}{A_{10}}+{A_1}{A_4}{A_6}{A_{11}}+{A_2}{A_3}{A_6}{A_{11}}+{A_2}{A_4}{A_6}{A_{10}} - {a_1}{h_4}{A_4}{A_{10}},$$

$${A_{19}}={\mathrm{i}}r{h_2}{A_6}{A_9} - {r^2}h_{2}^{2}{A_5}{A_{10}} - {r^2}h_{2}^{2}{A_6}{A_{11}}+{r^2}{h_2}{A_6}{A_8} - {\mathrm{i}}r{A_1}{A_6}{A_9},$$

$${A_{20}}=2{r^2}{h_2}{h_4}{a_1}{A_{10}} - {r^2}{h_4}{a_1}{A_1}{A_{10}} - {a_4}{A_1}{A_3}{A_{12}}+{a_4}{h_4}{A_3}{A_8} - {a_1}{h_4}{A_3}{A_{11}},$$

$${A_{21}}={a_1}{A_4}{A_{12}} - {A_4}{A_5}{A_8}+{A_1}{A_4}{A_5}{A_{11}}+{A_2}{A_3}{A_5}{A_{11}}+{A_2}{A_4}{A_5}{A_{10}},$$

$${A_{22}}={A_2}{A_4}{A_6}{A_{11}} - {a_4}{A_1}{A_4}{A_{12}} - {a_4}{A_2}{A_3}{A_{12}}+{a_4}{h_4}{A_4}{A_8} - {a_1}{h_4}{A_4}{A_{11}},$$

$${A_{23}}= - 2{a_1}{h_2}{r^2}{A_{12}}+{\mathrm{i}}r{h_2}{A_5}{A_9} - {r^2}h_{2}^{2}{A_5}{A_{11}}+{r^2}h_{2}^{2}{a_4}{A_{12}}+{a_1}{r^2}{A_1}{A_{12}},$$

$${A_{24}}={h_2}{r^2}{A_5}{A_8} - {\mathrm{i}}r{A_1}{A_5}{A_9} - {\mathrm{i}}r{A_2}{A_6}{A_9} - {a_1}{h_4}{r^2}{A_1}{A_{11}} - {a_1}{h_4}{r^2}{A_2}{A_{10}},$$

$${A_{25}}={\mathrm{i}}r{a_4}{h_4}{A_1}{A_9} - {a_4}{h_2}{h_4}{r^2}{A_8}+2{a_1}{h_2}{h_4}{r^2}{A_{11}} - {\mathrm{i}}r{a_4}{h_2}{h_4}{a_4}{A_9},$$

$${A_{26}}={A_2}{A_4}{A_5}{A_{11}} - {a_4}{A_2}{A_4}{A_{12}}+{a_1}{r^2}{A_2}{A_{12}} - {\mathrm{i}}r{A_2}{A_5}{A_9} - {r^2}{a_1}{h_4}{A_2}{A_{11}},\,\,\,\,\,{A_{27}}={\mathrm{i}}r{a_4}{h_4}{A_2}{A_9}\,.$$

The Eq. (18) is constituent as:

19 $${\mathrm{(}}{{\mathrm{D}}^{\mathrm{2}}} - k_{1}^{2}{\mathrm{)}}({{\mathrm{D}}^{\mathrm{2}}} - k_{2}^{2})\,({{\mathrm{D}}^{\mathrm{2}}} - k_{3}^{2})\,({{\mathrm{D}}^{\mathrm{2}}} - k_{4}^{2})\bar {u}\,(x)=0,$$

where $k_{n}^{2}\,\,(\,n=1,\,2,\,3,\,4\,)$ are the roots of the equation:

20 $${k^8} - {E_1} {k^6}+{E_2} {k^4} - {E_3} {k^2}+{E_4}=0.$$

The delimited result of Eq. (18) is

21 $$\bar {u}\,(x)=\sum\limits_{{n={\mathrm{1}}}}^{{\mathrm{4}}} {\,{N_n}} \,{\mathrm{exp(}} - {k_n}x{\mathrm{)}}$$

Similarly,

22 $$\bar {v}\,(x)=\sum\limits_{{n={\mathrm{1}}}}^{{\mathrm{4}}} {\,{H_{1n}}{N_n}} \,{\mathrm{exp(}} - {k_n}x{\mathrm{)}} - {G_0},$$

23 $$\bar {\theta }\,(x)=\sum\limits_{{n={\mathrm{1}}}}^{{\mathrm{4}}} {\,{H_{2n}}{N_n}} \,{\mathrm{exp(}} - {k_n}x{\mathrm{)}} - {G_1},$$

24 $$\bar {\psi }\,(x)=\sum\limits_{{n={\mathrm{1}}}}^{{\mathrm{4}}} {\,{H_{3n}}{N_n}} \,{\mathrm{exp(}} - {k_n}x{\mathrm{)}} - {G_2}$$

Using Eqs. (7), (12), (13) in Eq. (1), after that introduced Eqs. (21)–(24), we get

25 $${\bar {\sigma }_{xx}}(x)=\sum\limits_{{n={\mathrm{1}}}}^{{\mathrm{4}}} {{H_{4n}}\,{N_n}} \,{\mathrm{exp(}} - {k_n}x{\mathrm{)}}+{S_1}{\mathrm{,}}$$

26 $${\bar {\sigma }_{xy}}(x)=\sum\limits_{{n={\mathrm{1}}}}^{{\mathrm{4}}} {{H_{5n}}\,{N_n}} \,{\mathrm{exp(}} - {k_n}x{\mathrm{)}} - {S_2}{\mathrm{,}}$$

$$where,\,\,A_{{28}} = irA_{2} (a_{4} h_{4} - A_{5} ),A_{{29}} = A_{2} (A_{4} A_{5} - a_{1} h_{4} r^{2} ),A_{{30}} = A_{2} (a_{4} A_{4} - a_{1} r^{2} ),$$

$${H_{1n}}=\frac{{ir({h_2}k_{n}^{2}+{A_2} - {A_1}k_{n}^{2})}}{{{r^2}{h_2}{k_n}+{A_3}k_{n}^{3} - {A_4}{k_n}}},\,\,\,{H_{2n}}=\frac{{{a_1}{h_4}k_{n}^{2} - ({A_5} - {A_6}k_{n}^{2})\,({A_1}k_{n}^{2} - {A_2}) - {\mathrm{i}}r{k_n}({a_1}{h_4} - {h_2}(({A_5} - {A_6}k_{n}^{2})\,{H_{1n}})}}{{{k_n}({A_5} - {A_6}k_{n}^{2}) - {a_4}{h_4}{k_n}}},$$

$${H_{3n}}=\frac{{{a_1}{k_n} - {\mathrm{i}}r{a_1}{H_{1n}}+{a_4}{H_{2n}}}}{{{A_5} - {A_6}k_{n}^{2}}},\,\,\,{H_{4n}}=\frac{{(ir{h_5}{H_{1i}} - {h_1}{k_i}+{h_4}{H_{3i}} - {H_{2i}})}}{{(1 - {\varepsilon ^2}k_{i}^{2}+{\varepsilon ^2}{r^2})}},\,\,\,\,{H_{5n}}=\frac{{(ir{h_3} - {h_3}{k_i}{H_{1i}})}}{{(1 - {\varepsilon ^2}k_{i}^{2}+{\varepsilon ^2}{r^2})}},$$

$${S_1}=\frac{{{A_{13}}{Q_0}{V_0}({A_{29}} - {\mathrm{i}}r{h_5}{A_{28}} - {h_4}{A_{30}})}}{{{A_{14}}{E_4}(1+{\varepsilon ^2}{r^2})}},\,\,\,\,\,\,{S_2}=\frac{{{h_3}{A_{13}}{A_{28}}{Q_0}{V_0}}}{{{A_{14}}{E_4}(1+{\varepsilon ^2}{r^2})}},\,\,\,\,\,{G_0}=\frac{{{A_{13}}{A_{28}}{Q_0}{V_0}}}{{{A_{14}}{E_4}}},$$

$${G_1}=\frac{{{A_{13}}{A_{29}}{Q_0}{V_0}}}{{{A_{14}}{E_4}}},\,\,\,\,\,\,\,\,{G_2}=\frac{{{A_{13}}{A_{30}}{Q_0}{V_0}}}{{{A_{14}}{E_4}}}.$$

## Boundary conditions

Two-dimensional deformation in the $xy$-plane is considered, and the position within the medium is defined as:

27 $$\chi =\left\{ {(x,y,z):\,\,0 \leqslant x<\infty ,\,0<y<\infty ,\,\, - \infty <z<\infty } \right\}.$$

In the physical problem, we should suppress the positive exponentials that are unbounded at infinity. To acquire the result of the head, we believe the leading boundary situation of the surface at $\,x=0:$.

- Thermal Condition: The half-space is subjected to a thermal shock at the boundary, resulting in:
  28 $$\theta ={f_1}.$$
- The condition of the voids volume fraction fields constant in *x* direction:
  29 $$\psi =0.$$
- The normal stress (mechanical stress) at the boundary is a constant force, expressed as:
  30 $${\sigma _{xx}}= - {f_2}\,{e^{{\mathrm{i}}\,r\,y - mt}}.$$
- The tangential stress is zero, meaning the boundary is stress-free:
  31 $${\sigma _{xy}}=0.$$

where ${f_1},\,{f_2}$ are constants.

By artifact Eqs. (23)–(26) in Eqs. (28)–(31), we obtain

32 $$\sum\limits_{{n={\mathrm{1}}}}^{{\mathrm{4}}} {\,{H_{2n}}{N_n}} \,={f_1}+{G_1},\,\,\,\,\,\,\sum\limits_{{n={\mathrm{1}}}}^{{\mathrm{4}}} {\,{H_{3n}}{N_n}} \,={G_2},\,\,\,\,\,\sum\limits_{{n={\mathrm{1}}}}^{{\mathrm{4}}} {\,{H_{4n}}{N_n}} \,= - {f_2} - {S_1},\,\,\,\,\,\,\,\sum\limits_{{n={\mathrm{1}}}}^{{\mathrm{4}}} {\,{H_{5n}}{N_n}} \,={S_2}\,.$$

Using the matrix, the resolution to Eq. (32) is:

33 $$\left( {\begin{array}{*{20}{c}} {{N_1}} \\ {{N_2}} \\ {{N_3}} \\ {{N_4}} \end{array}} \right)={\left( {\begin{array}{*{20}{c}} {{H_{21}}}&{{H_{22}}}&{{H_{23}}}&{{H_{24}}} \\ {{H_{31}}}&{{H_{32}}}&{{H_{33}}}&{{H_{34}}} \\ {{H_{41}}}&{{H_{42}}}&{{H_{43}}}&{{H_{44}}} \\ {{H_{51}}}&{{H_{52}}}&{{H_{53}}}&{{H_{54}}} \end{array}} \right)^{ - 1}}\left( {\begin{array}{*{20}{c}} {{f_1}+{G_1}} \\ {{G_2}} \\ { - {f_2} - {S_1}} \\ {{S_2}} \end{array}} \right).$$

## Particular cases and special cases of thermoelastic theory

(i) The corresponding equations for a porous thermoelastic half-space subjected to a moving heat source from the above-mentioned cases by taking $\varepsilon =0$.
(ii) The corresponding equations for a nonlocal porous thermoelastic half-space from the above-mentioned cases by taking ${Q_0}=0$.
(iii) The corresponding equations for a nonlocal thermoelastic half-space subjected to a moving heat source from the above-mentioned cases by taking $\beta ,\,b,\,{\alpha _1},\,{\alpha _2},\,{\alpha _3},\,{\alpha _4}=0$.
(iv) Equations of the 3PHL model when, $K,{\tau _\theta },\,{\tau _q},\,{\tau _\nu }>0,$ and the solutions are always (exponentially) stable if $\frac{{2K{\tau _\theta }}}{{{\tau _q}}}\,>\tau _{\nu }^{*}>{K^*}{\tau _q}$ as in Quintanilla and Racke^37^.
(v) Equations of the G-N II theory when, $K={\tau _\theta }=\,{\tau _q}=\,{\tau _\nu }=0$.
(vi) Equations of the G-N III theory when, ${\tau _\theta }=\,{\tau _q}=\,{\tau _\nu }=0$.

## Discussion of numerical results

To compare the outcomes via the 3PHL and G-N III, we consider the numerical outcomes. for the physical constants as Said et al.^23^:

$$\tau _{\theta } = 0.07s,\tau _{\upsilon } = 0.03s,m_{0} = 0.3,\xi = - 0.4,T_{0} = 293K,\,m = m_{0} + i\xi ,\beta = 2 \times 10^{5} N.m^{{ - 2}} ,$$

$$C_{E} = 383.1\,\,J\,.kg^{{ - 1}} .\,K^{{ - 1}} \,,\,\,\,Q_{0} = 9\, \times 10^{9} \,K\,,\,\,\,f_{2} = 3.5,\,\,\,\alpha _{4} = 1.753\, \times 10^{{ - 5}} \,N\,.\,m^{{ - 2}} \,,\,\,\,y = 1.3,$$

$$\alpha _{t} = 2.78\, \times 10^{{ - 2}} K^{{ - 1}} ,\,\,\,\,K^{*} = 386w\,.\,m^{{ - 1}} .\,K^{{ - 1}} .s^{{ - 1}} ,\,\,\,\,K = 386w\,.\,m^{{ - 1}} .\,K^{{ - 1}} ,\,\,\,b = 1.6\, \times 10^{7} \,N.m^{{ - 2}} ,\,$$

$$\alpha _{1} = 1.47\, \times 10^{5} N.m^{{ - 2}} ,\,\,\,\alpha _{2} = 7.78\, \times 10^{{ - 10}} N.m^{{ - 2}} ,\,\,\alpha _{3} = 2\, \times 10^{{10}} N.m^{{ - 2}} ,\,\,\,v_{0} = 100\,m.s^{{ - 1}} ,\,\,f_{1} = 0.9.$$

 The computations were carried out for non-dimensional time $t = 0.005$. The reasoned physical variables are shown in graphs 1–10.

### The impact of locality

**Fig. 1:**
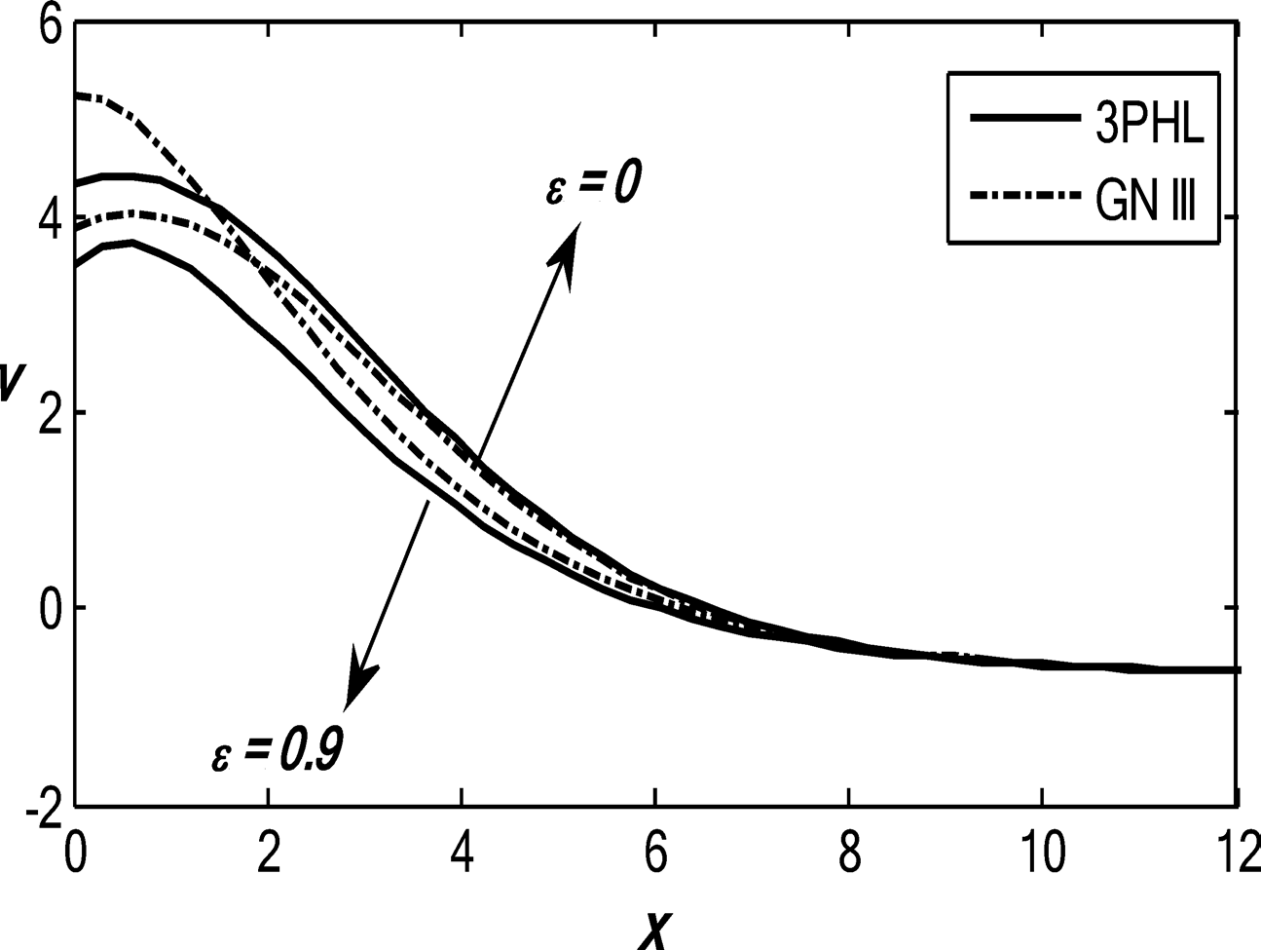
Variation of the dimensionless vertical displacement *v* with the dimensionless depth *x* based on the 3PHL and GN-III models, for different values of the nonlocal parameter $(\varepsilon =0.9,\,\,0)$, in the presence of a moving heat source.

**Fig. 2:**
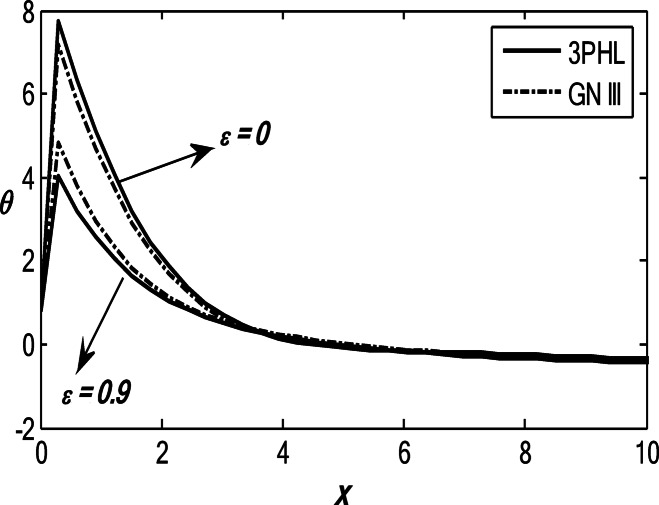
Variation of the dimensionless temperature $\theta$ with the dimensionless depth *x* based on the 3PHL and GN-III models, for different values of the nonlocal parameter $(\varepsilon =0.9,\,\,0)$ in the presence of a moving heat source.

**Fig. 3:**
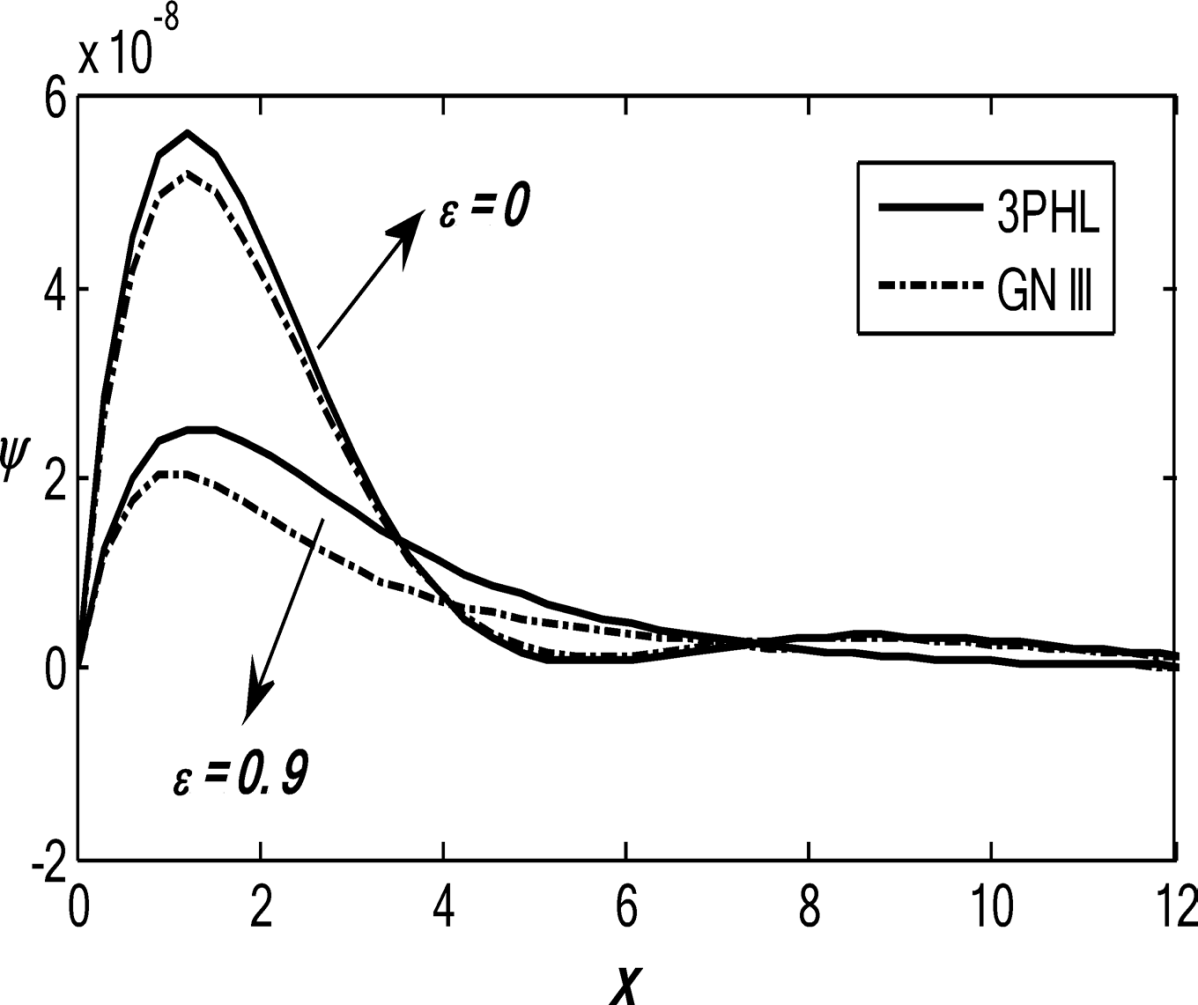
Variation of the dimensionless volume fraction field $\psi$ with the dimensionless depth *x* based on the 3PHL and GN-III models, for different values of the nonlocal parameter $(\varepsilon =0.9,\,\,0)$, in the presence of a moving heat source.

**Fig. 4:**
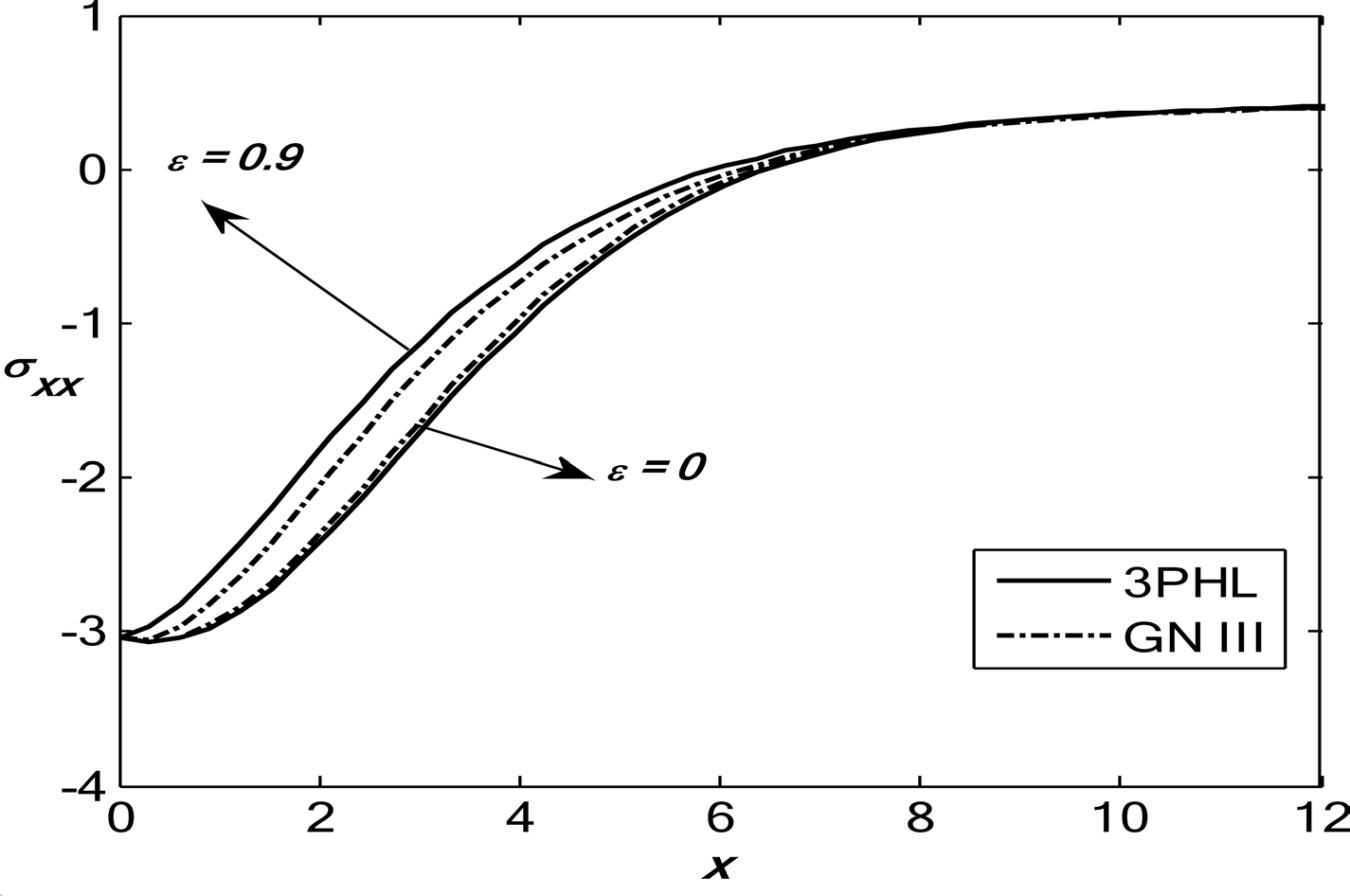
Variation of the dimensionless stress component ${\sigma _{xx}}$ with the dimensionless depth *x* based on the 3PHL and GN-III models, for different values of the nonlocal parameter $(\varepsilon =0.9,\,\,0)$ in the presence of a moving heat source.

**Fig. 5:**
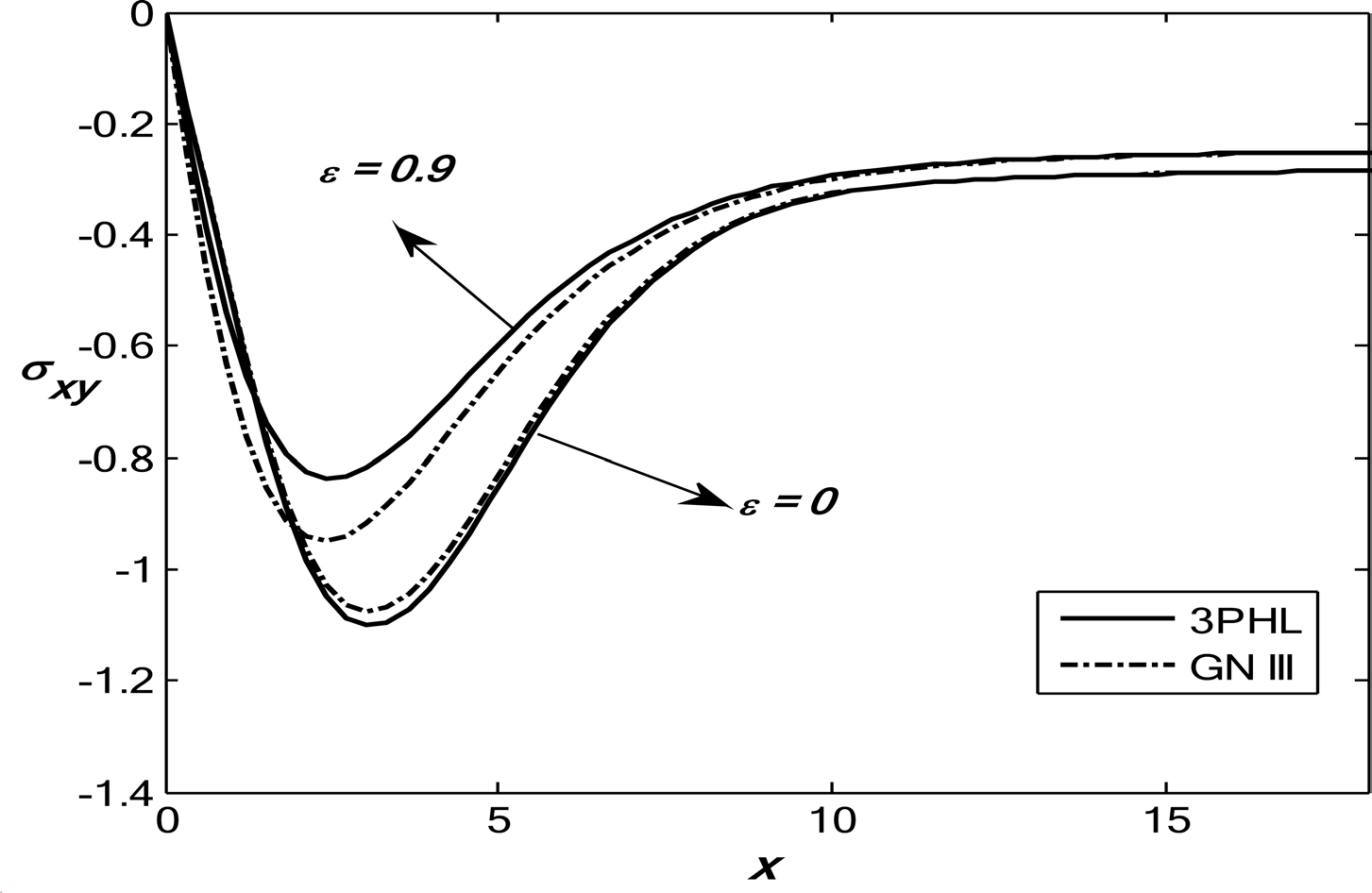
Variation of the dimensionless stress component ${\sigma _{xy}}$ with the dimensionless depth *x* based on the 3PHL and GN-III models, for different values of the nonlocal parameter $(\varepsilon =0.9,\,\,0)$ in the presence of a moving heat source.

Figures 1, 2, 3, 4 and 5 exhibit the vertical displacement *v*, the stress components ${\sigma _{xx}},\,{\sigma _{xy}}$, thermodynamic temperature distributions $\theta ,$ and change in volume fraction field $\psi$ in the lack and existence of the locality. Figure 1 represents that the alteration of *v* starts with positive values. *v* decreases in the scope $0\,\, \le x\, \le 12$. The locality causes depreciating to values of *v*. Figure 2 displays the alteration of $\theta$starts with positive values and satisfies the boundary conditions. Values of $\theta$ gain to a maximum value, and then drop-off. The locality causes dwindling to values of $\theta$. Also, we noted that in the presence of non-locality, the temperature values are lower than those in the local case. This is due to the enhanced diffusion of thermal energy induced by nonlocal effects, which smooth out sharp thermal gradients. Figure 3 dramas the alteration of $\psi$ starts with zero magnitude and conforms to the boundary situation. In the existence of the locality, $\psi$ gains to a maximum value, and then drops off. In the lack of locality, $\psi$ gains to a maximum magnitude, then drops off to a minimum magnitude, and endmost takes a wave extension. This figure demonstrates that the change in the volume fraction field exhibits oscillatory behavior with depth, which becomes more regular when nonlocality is taken into account. The nonlocal interactions suppress localized mechanical responses associated with the porous microstructure, leading to reduced oscillation amplitudes and enhanced mechanical stability of the medium. Figure 4 inserts that the fluctuation of ${\sigma _{xx}}$ starts out with negative values and adjusts the boundary situation. It increases in the limit $0 \leqslant x \leqslant 12.$ The locality causes growth to the values of ${\sigma _{xx}}$. The presence of nonlocality increases the absolute magnitude of the stress compared to the local case. This reflects the redistribution of internal stresses due to long-range interactions, which modifies the stress transfer mechanism relative to classical elasticity. Figure 5 interprets that the modification of ${\sigma _{xy}}$ starts with a zero magnitude and satisfies the boundary conditions. ${\sigma _{xy}}$ starts depreciating to reach its minimum value and then increases. The locality causes flourishing to values of ${\sigma _{xy}}$.

### The impact of moving heat source

**Fig. 6:**
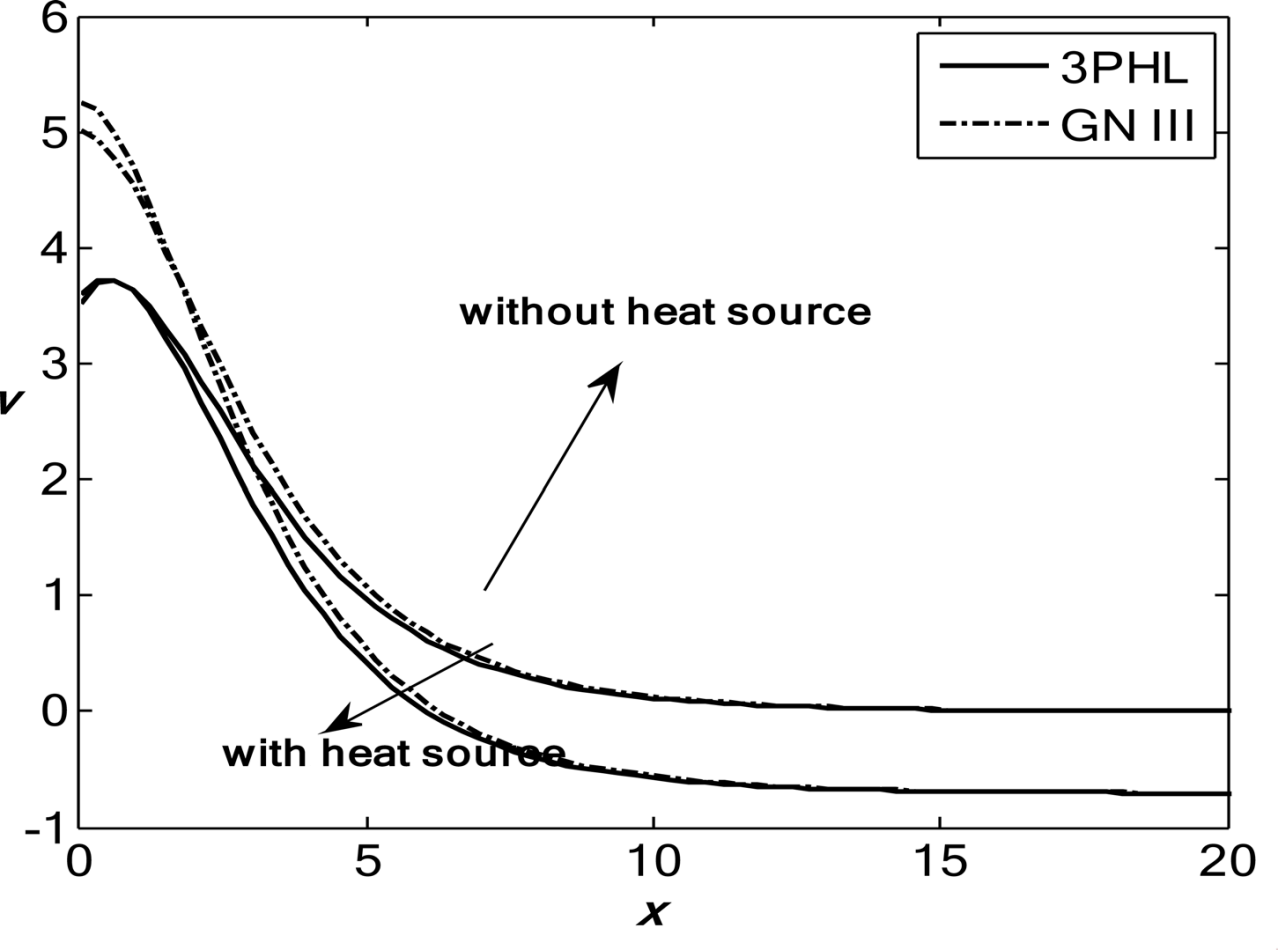
Variation of the dimensionless displacement component *v* with the dimensionless depth *x* based on the 3PHL and GN-III models, in the presence and absence of a moving heat source ${Q_0}$.

**Fig. 7:**
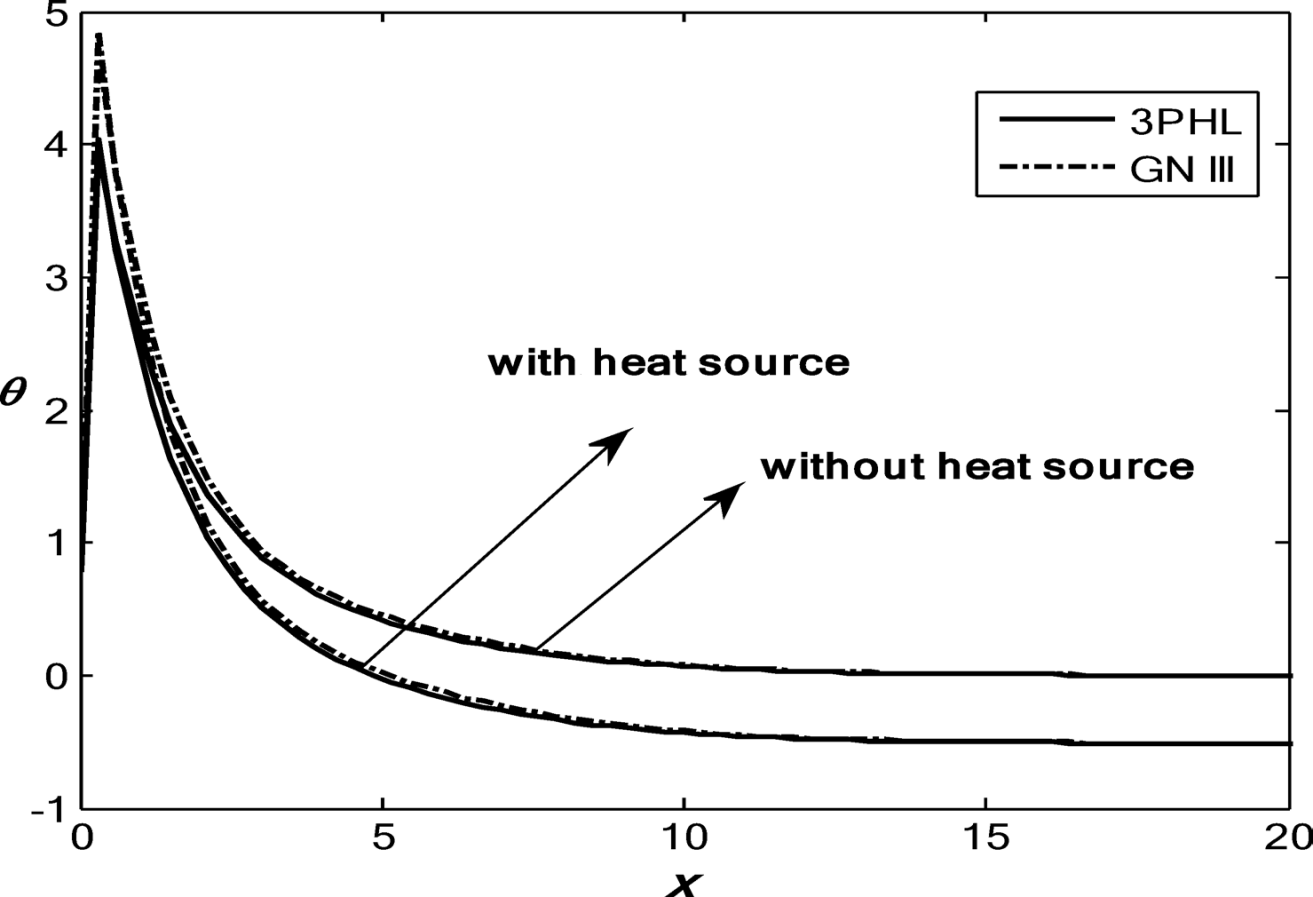
Variation of the dimensionless temperature $\theta$ with the dimensionless depth based on the 3PHL and GN-III models, in the presence and absence of a moving heat source.

**Fig. 8:**
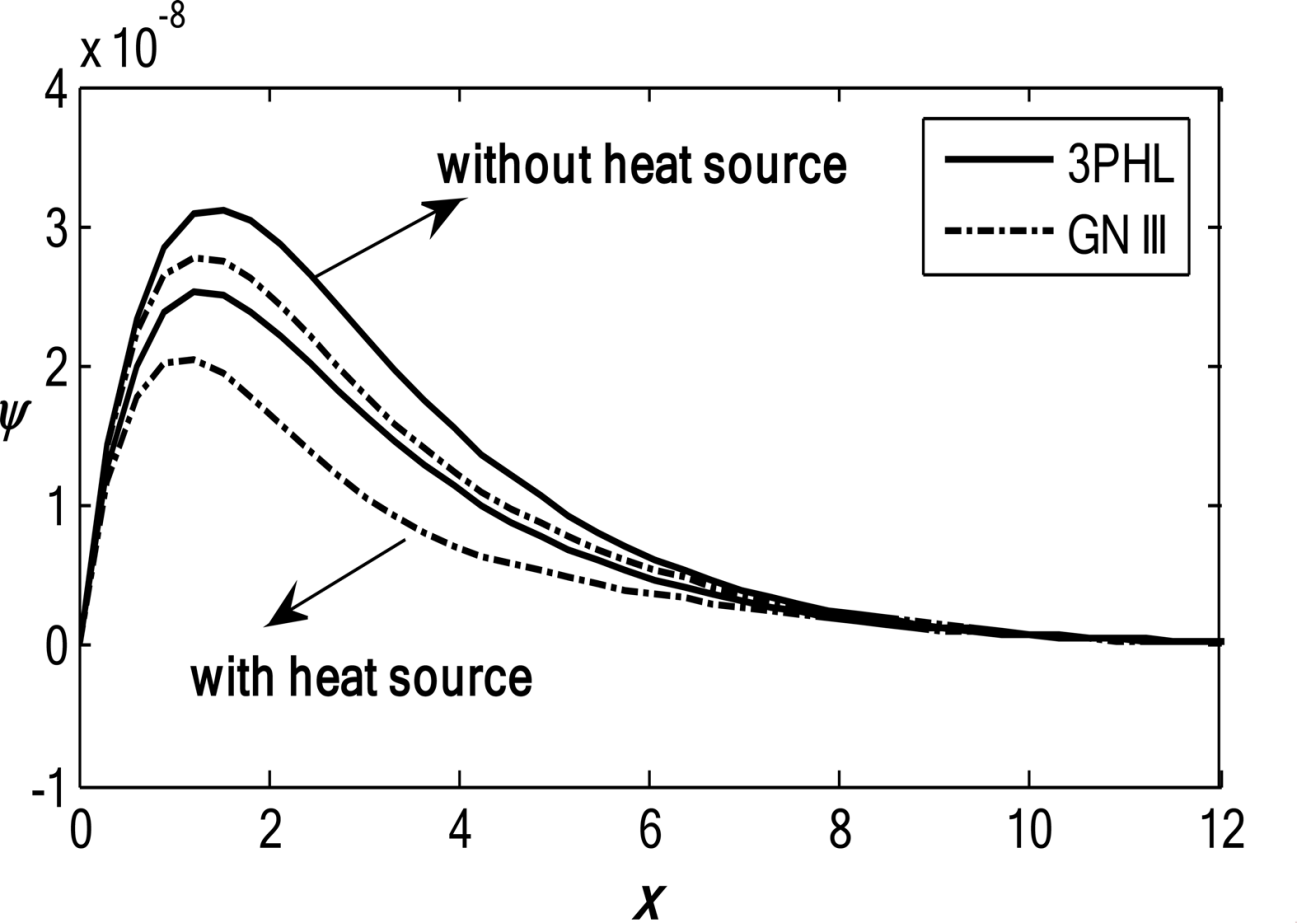
Variation of the dimensionless volume fraction field $\psi$ with the dimensionless depth *x* based on the 3PHL and GN-III models, in the presence and absence of a moving heat source ${Q_0}$.

**Fig. 9:**
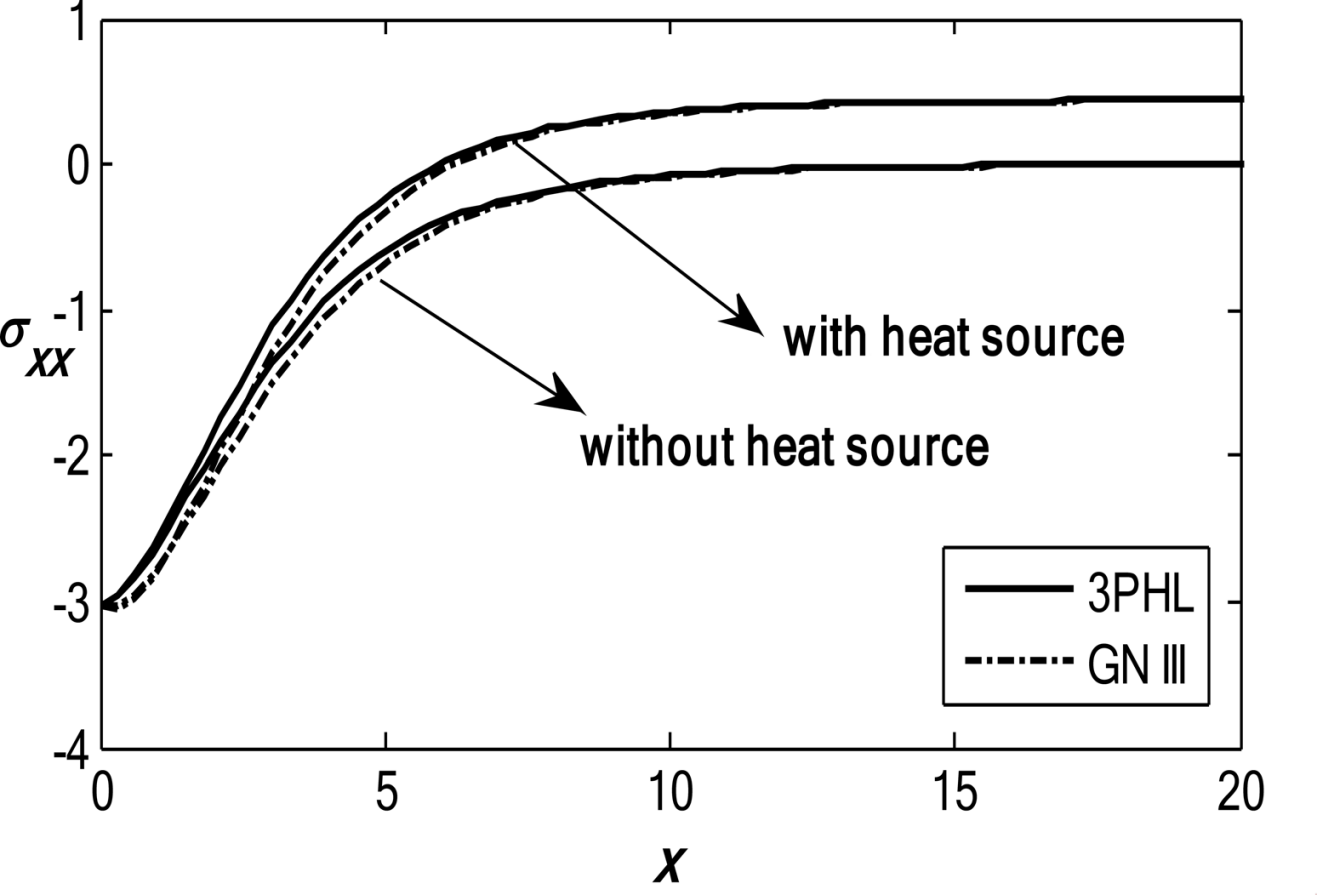
Variation of the dimensionless stress component $\sigma_{xx}$ with the dimensionless depth based on the 3PHL and GN-III models, in the presence and absence of a moving heat source.

**Fig. 10:**
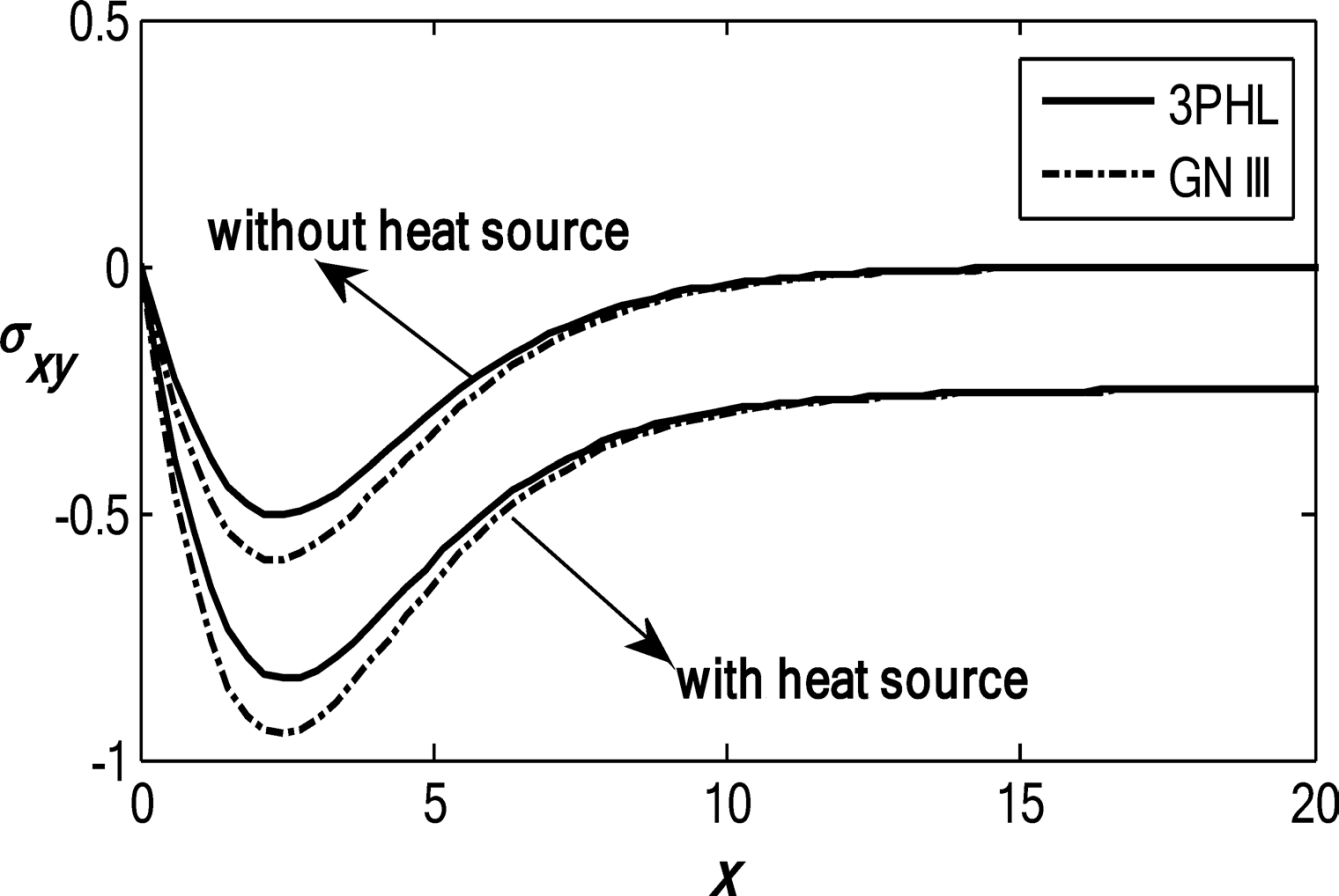
Variation of the dimensionless stress component $\sigma_{xy}$ with the dimensionless depth based on the 3PHL and GN-III models, in the presence and absence of a moving heat source.

Figures 6, 7, 8, 9 and 10 exhibit the vertical displacement *v*, the stress components ${\sigma _{xx}},\,{\sigma _{xy}}$, thermodynamic temperature distributions $\theta ,$ and change in volume fraction field $\psi$ in the deficiency and proximity of a moving internal heat source. Figure 6 illustrates the variation of the dimensionless vertical displacement *v* with the dimensionless depth *x* in the presence and absence of a moving heat source for both the 3PHL and G-N III models. The displacement starts with positive values at the surface for all cases and gradually decreases with increasing depth due to the attenuation of thermal effects inside the medium. In the absence of a moving heat source, the displacement values are higher than those obtained in the presence of the heat source. The moving heat source redistributes the thermal loading within the medium, thereby reducing the net displacement and, at larger depths, may even reverse its direction. Moreover, the 3PHL model exhibits a more damped displacement response compared to the G-N III model. Figure 7 dramas the alteration of $\theta$ starts with positive magnitudes and obeys the boundary conditions. The temperature attains its maximum value at the surface due to the applied thermal shock and decreases rapidly with depth as heat propagates into the medium. Figure 8 depicts the variation of the dimensionless volume fraction field $\psi$ with the dimensionless depth *x*. The volume fraction starts from zero at the surface, satisfying the boundary conditions, then increases to a maximum value at a finite depth before gradually decaying as the depth increases. The magnitude of the volume fraction field in the absence of a moving heat source is greater than that observed in the presence of the heat source, indicating that the motion of the heat source suppresses the amplitude of porosity-related variations by redistributing the thermal influence throughout the medium. In addition, the 3PHL model exhibits a more pronounced response compared to the G-N III model. Figure 9 inserts that the variance of ${\sigma _{xx}}$ begins with negative magnitude and adjusts the boundary conditions. In the presence of a moving heat source, the values of ${\sigma _{xx}}$ are higher (less negative) than those in the absence of the heat source. This behavior results from the redistribution of thermal gradients caused by the moving heat source, which alleviates the concentration of compressive stresses near the surface. Figure 10 interprets that the alteration of ${\sigma _{xy}}$ starts with a zero magnitude and adjusts the boundary conditions. The results indicate that the presence of a moving heat source increases the magnitude of the shear stress (in absolute value) compared to the case without a heat source. This reflects the role of the moving heat source in generating non-uniform thermal gradients that enhance shear deformation within the porous medium, before these effects dissipate at larger depths.

**Fig. 11:**
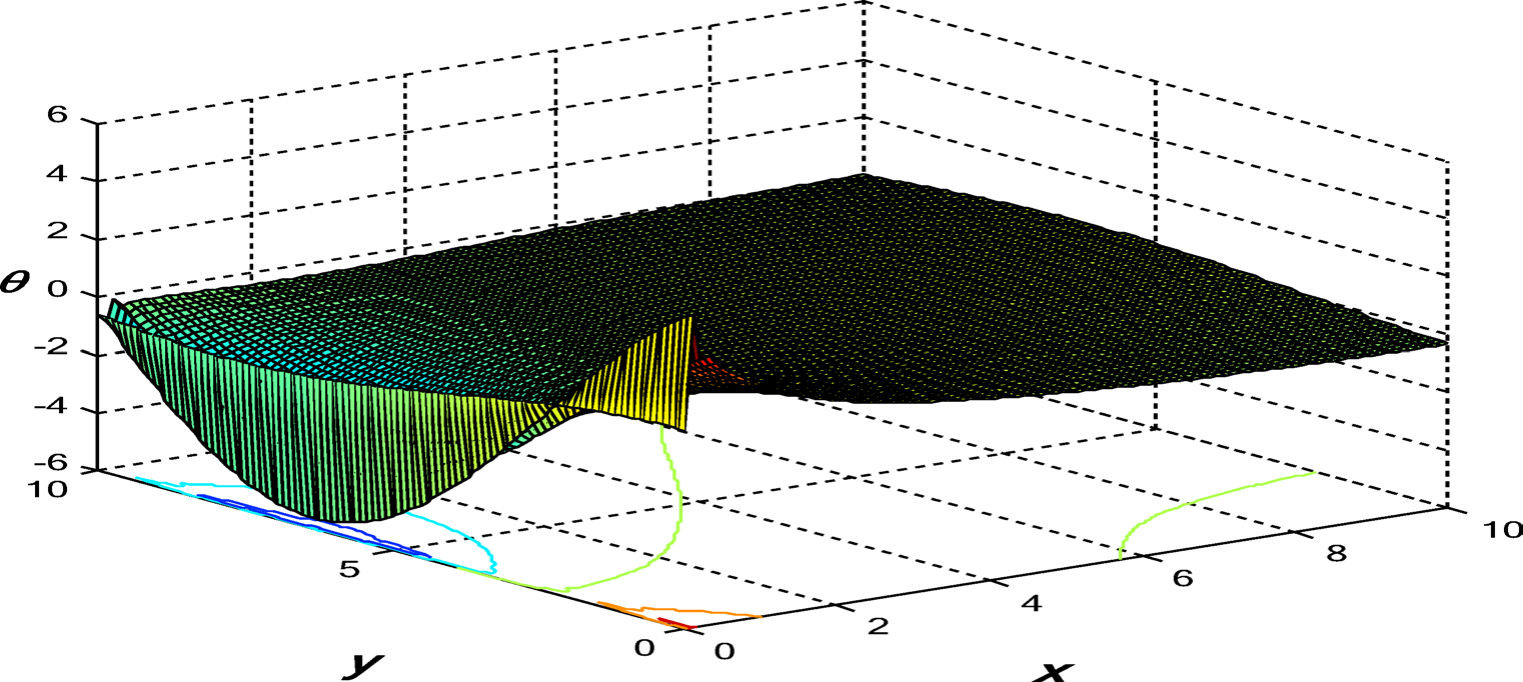
Variation of the dimensionless thermodynamics’ temperature $\theta$ in the context of 3PHL model.

**Fig. 12:**
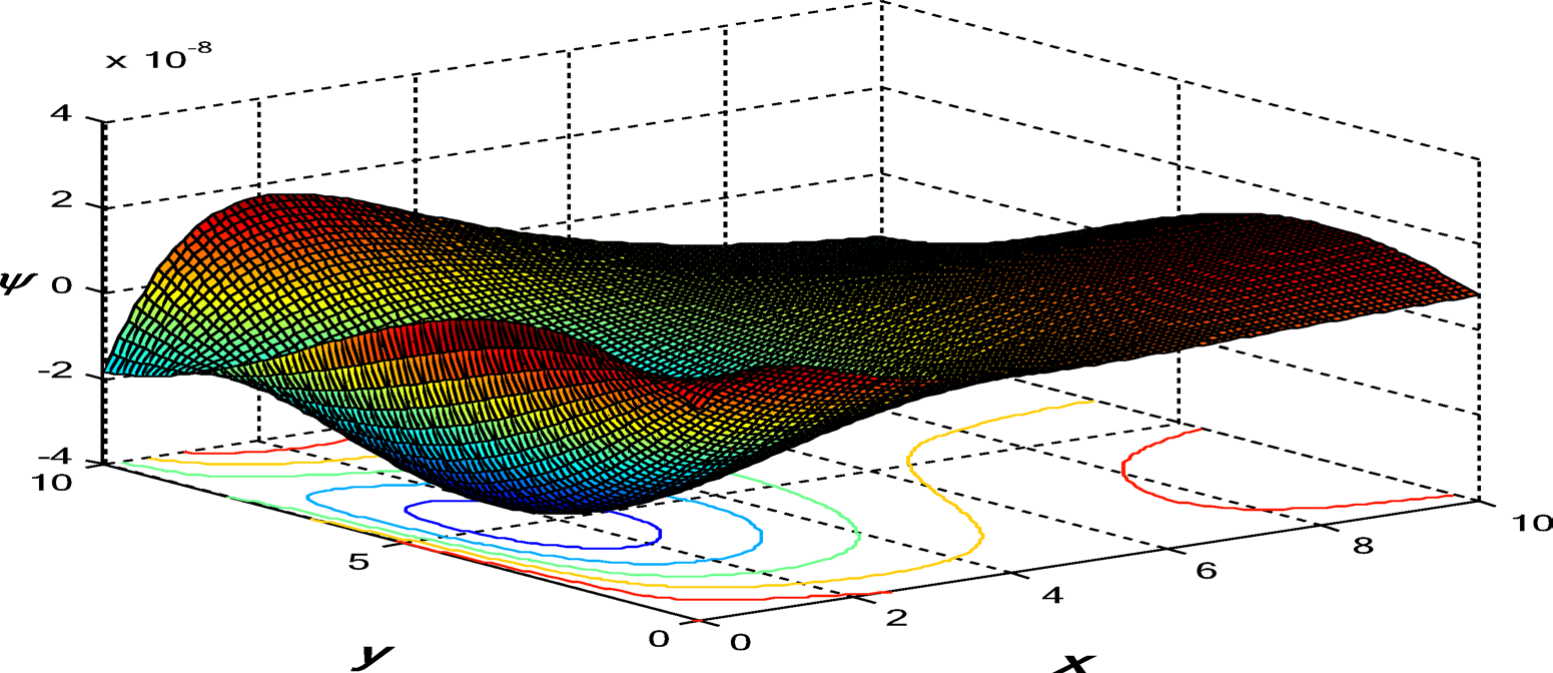
Variation of change in volume fraction field $\psi$ in the context of 3PHL model.

**Fig. 13:**
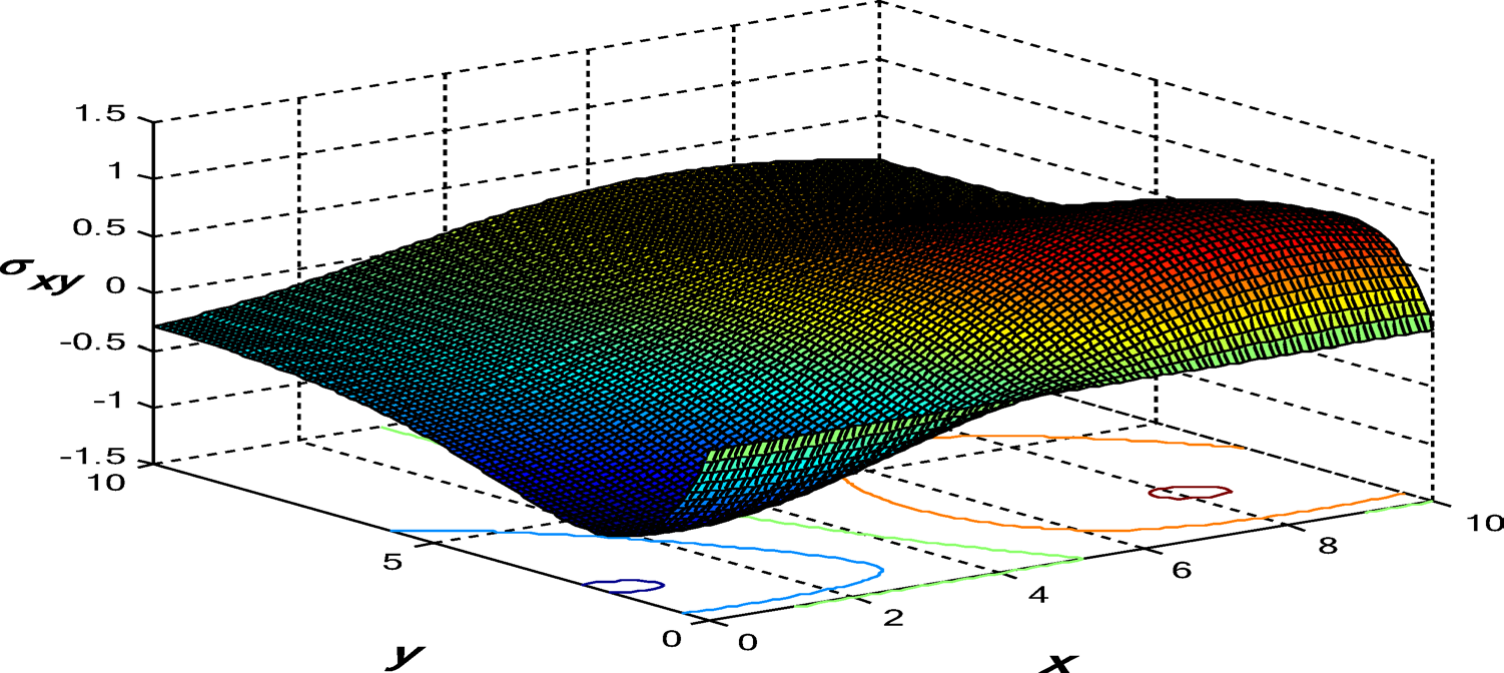
Distribution of stress component ${\sigma _{xy}}$ in the context of 3PHL model.

Figures 11, 12 and 13 show the 3D surface curves for $\theta ,\,\psi$ and ${\sigma _{xy}}.$ The effects of internal heat source on wave propagation in a nonlocal thermoelastic medium with pores are explained according to the 3PHL model. These figures are very helpful in discussing the dependence of these physics’ fields on the vertical component of distance.

The present numerical results obtained in this study are also supported by comparisons with previously published numerical investigations on nonlocal and generalized thermoelastic media subjected to moving heat sources. In particular, the attenuation of displacement and stress amplitudes due to nonlocal effects observed here is consistent with the numerical findings reported by Bayones et al.^10^ and Das et al.^33^. Similarly, the influence of a moving heat source on temperature and stress distributions agrees well with the trends obtained by Ailawalia and Singla^28^ and Abbas^29^ using generalized and dual-phase-lag thermoelastic models. Moreover, the smoothing of thermal gradients and redistribution of stresses predicted in the present study are in qualitative agreement with results obtained from fractional-order and space–time nonlocal thermoelastic analyses, confirming the physical soundness and numerical reliability of the proposed model.

## Conclusion

This study examined the propagation of thermoelastic waves in a nonlocal porous half-space subjected to a moving heat source, within the frameworks of the 3PHL heat conduction model and Green–Naghdi type III theory. Closed-form analytical solutions for displacement, temperature, stress, and volume fraction fields were derived using normal mode analysis, enabling a detailed investigation of the coupled thermo-elastic behavior. It noticed that:

- The results demonstrate that nonlocal effects significantly attenuate displacement and stress amplitudes, reflecting the influence of long-range interactions, whereas porosity predominantly affects temperature distribution and volume fraction variations, underscoring the role of microstructural characteristics.
- Phase-lag parameters were found to introduce appreciable thermal delays under dynamic loading, substantially modifying wave behavior near the boundary. The originality of this work lies in its unified treatment of nonlocality, porosity, and moving thermal loading, extending beyond prior studies that focus on local theories or stationary heat sources.
- Comparative analysis between the 3PHL model and Green–Naghdi type III theory under identical conditions highlights distinct differences in thermal response, displacement, and stress distribution.
- It is clear that the moving heat source plays a significant role on all physical quantities as it causes decrease of magnitudes of most physical quantities.

These findings provide valuable insight into thermoelastic wave propagation in porous media under dynamic thermal conditions and have potential applications in advanced engineering systems, including laser material processing, additive manufacturing, and thermal barrier design.

## Data Availability

The data sets used and/or analyzed during the current study available from the corresponding author on reasonable request.

## List of symbols

$\sigma_{ij}$: Components of stress tensor
$e_{ij}$: Components of strain tensor
$$T$$: Absolute temperature
$T_{0}$: Reference temperature such that $\left| {(T - T_{0} )/T_{0} } \right| < 1$ , $\theta_{0} = T - T_{0}$
$\tau_{\theta }$: Phase lag of temperature gradient
$\tau_{q}$: Phase lag of heat flux
$\tau_{\nu }$: Thermal gradient of displacement with phase lag, $\tau_{\nu }^{*} = K + K^{*} \tau_{\nu }$**.**
$\beta ,\,b,\,\alpha_{1} ,\alpha_{2} ,\,\alpha_{3} ,\,\alpha_{4}$: Porosity-dependent material constants
$u_{i}$: Components of displacement
$$\lambda ,\,\mu$$: Lame’s constant
$$\delta_{ij}$$: Kronecker’s delta
$K^{*}$: Coefficient of thermal conductivity
$K$: Constant of additional material
$$\rho$$: Density
$C_{E}$: Specific heat at fixed strain
*i*: Imaginary, $${\mathrm{i}} = \sqrt { - 1}$$
$$r$$: Wave number
$m$: Constant is complex
$Q$: Moving internal heat source
$Q_{0}$: Magnitude of an internal heat source
$v_{0}$: Velocity of moving internal heat source
$\alpha_{t}$: Linear thermal expansion coefficient, $\gamma = (\,3\lambda + 2\mu \,)\alpha_{t} ,\,\,$
$\psi$: Volume fraction field change of voids
$\varepsilon = a_{0} \,e_{0}$: $a_{0} \,$ an internal characteristic of length and $\,e_{0}$ constant of a material
